# Engineering the Chloroplast Targeted Malarial Vaccine Antigens in *Chlamydomonas* Starch Granules

**DOI:** 10.1371/journal.pone.0015424

**Published:** 2010-12-15

**Authors:** David Dauvillée, Stéphane Delhaye, Sébastien Gruyer, Christian Slomianny, Samuel E. Moretz, Christophe d'Hulst, Carole A. Long, Steven G. Ball, Stanislas Tomavo

**Affiliations:** 1 Centre National de la Recherche Scientifique, CNRS UMR 8576, UGSF, Université des Sciences et Technologies de Lille, Villeneuve d'Ascq, France; 2 Center for Infection and Immunity of Lille, CNRS UMR 8204, INSERM U 1019, Institut Pasteur de Lille, Université Lille Nord de France, Lille, France; 3 Laboratoire de Physiologie Cellulaire, INSERM U 1003, Université des Sciences et Technologies de Lille, Villeneuve d'Ascq, France; 4 Laboratory of Malaria and Vector Research, National Institute of Allergy and Infectious Disease, National Institutes of Health, Rockville, Maryland, United States of America; University of California Merced, United States of America

## Abstract

**Background:**

Malaria, an Anopheles-borne parasitic disease, remains a major global health problem causing illness and death that disproportionately affects developing countries. Despite the incidence of malaria, which remains one of the most severe infections of human populations, there is no licensed vaccine against this life-threatening disease. In this context, we decided to explore the expression of *Plasmodium* vaccine antigens fused to the granule bound starch synthase (GBSS), the major protein associated to the starch matrix in all starch-accumulating plants and algae such as *Chlamydomonas reinhardtii*.

**Methods and Findings:**

We describe the development of genetically engineered starch granules containing plasmodial vaccine candidate antigens produced in the unicellular green algae *Chlamydomonas reinhardtii.* We show that the C-terminal domains of proteins from the rodent *Plasmodium* species, *Plasmodium berghei* Apical Major Antigen AMA1, or Major Surface Protein MSP1 fused to the algal granule bound starch synthase (GBSS) are efficiently expressed and bound to the polysaccharide matrix. Mice were either immunized intraperitoneally with the engineered starch particles and Freund adjuvant, or fed with the engineered particles co-delivered with the mucosal adjuvant, and challenged intraperitoneally with a lethal inoculum of *P. Berghei.* Both experimental strategies led to a significantly reduced parasitemia with an extension of life span including complete cure for intraperitoneal delivery as assessed by negative blood thin smears. In the case of the starch bound *P. falciparum* GBSS-MSP1 fusion protein, the immune sera or purified immunoglobulin G of mice immunized with the corresponding starch strongly inhibited *in vitro* the intra-erythrocytic asexual development of the most human deadly plasmodial species.

**Conclusion:**

This novel system paves the way for the production of clinically relevant plasmodial antigens as algal starch-based particles designated herein as amylosomes, demonstrating that efficient production of edible vaccines can be genetically produced in *Chlamydomonas*.

## Introduction

Malaria is a mosquito-borne disease and remains a major global health problem causing illness and death that disproportionately affects developing countries. The worldwide incidence of malaria is estimated by the Word Health Organization to be approximately 300 to 500 million clinical cases annually, with one million deaths, the majority of which are young children [1]–[4]. Of particular concern, the emergence of insecticide-resistant mosquito vectors and multi-drug resistant parasites has contributed to resurgences of the disease. Therefore, malaria control is a continuous battle that requires long-term sustainability and commitment. The development of a vaccine that reduces morbidity and mortality would be a valuable new tool in the fight against malaria. *Plasmodium falciparum* causes the most severe form of the disease [5], [6]. Infection begins when malaria sporozoites are injected by mosquito into the host and within minutes parasites invade hepatocytes, where they multiply and differentiate into the next stage. The emerging merozoites invade red blood cells leading to clinical illness [7]. The most advanced vaccine candidate, designated RTS,S/AS02A, is based on the major sporozoite surface antigen. However, this candidate vaccine, currently in Phase 3 clinical trials, has shown only 30–65% efficiency in field studies [8] and a vaccine with higher levels of protection is still sought. Over time, people living in malaria-endemic areas develop immunity to clinical disease caused by *P. falciparum* and IgG from immune adults has been shown to reduce parasite density and clinical symptoms when administered to children with clinical malaria [9]–[11]. Thus, proteins expressed during the blood-stage of the life cycle are good candidates for inclusion in a vaccine [12], [13], as a blood-stage vaccine would reduce or prevent severe illness and complications of the disease.

In this context, we decided to explore the expression of *Plasmodium* vaccine antigens fused to the granule bound starch synthase (GBSS), the major protein associated to the starch matrix in all starch accumulating plants and algae [14], [15]. Starch-bound proteins are known to remain stable for years within the polysaccharide matrix purified from plants and algae. Starch can be easily purified from plants and algae by straightforward sedimentation procedures that in some systems do not even require centrifugation. In addition, cereal starch, with its role in human and animal diets, represents an approved source for the production of glucose for injection in humans. As a first approach to the use of recombinant polysaccharide particles for vaccine production, we focussed our efforts on the production of transgenic starch from chloroplasts of the green algae *Chlamydomonas reinhardtii*. We chose this system because of the ease and unparalleled speed with which constructs can be introduced and proteins expressed and correctly targeted to the chloroplast and polysaccharide granule. In addition, expression of recombinant vaccine antigens into starch granules localized into the *Chlamydomonas* chloroplasts would avoid protein *N*-glycosylation, a post-translational modification that seems to be generally absent in *Plasmodium falciparum*. Moreover, starch metabolism has been investigated in great detail in this system through genetic dissection of mutants allowing optimization of starch granule protein content and polysaccharide structure [16]–[18]. To evaluate this novel system, we have chosen two well-studied *Plasmodium* vaccine candidates, MSP1 [19], [20] and AMA1 [21], [22], which are thought to be involved in invasion of human red blood cells. The biosynthesis, purification, characterization, and immunologic properties of starch-stored clinically relevant antigens produced in *Chlamydomonas reinhardtii* chloroplast are described.

## Results and Discussion

### Genetic engineering of *Chlamydomonas* vectors expressing transgenic starch bound plasmodial antigens

**Figure 1:**
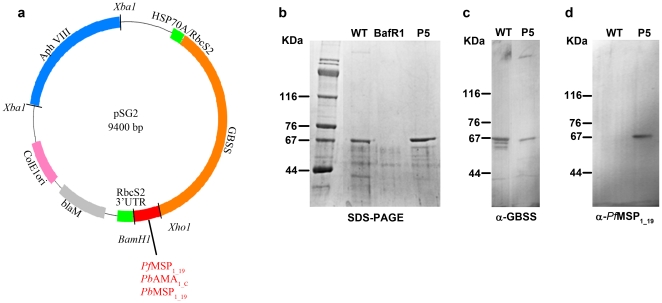
Anti-malaria vaccine strategy and antigen expression in *C. reinhardtii*. (**a**) Map of the vector used for *C. reinhardtii* transformation and expression. The plasmid of 9.4 kb, designated pSG2, carries the genomic region of 3 kb containing all introns and exons necessary for coding the *C. reinhardtii* granule bound starch synthase (GBSS). The *XhoI* and *BamHI* restriction sites at the end of the truncated GBSS gene have been utilized for cloning in-frame cDNAs encoding the C-terminal domain of *P. berghei* major surface protein 1 (MSP_1-19_); *P. berghei* apical major antigen (AMA_1-C_) and *P. falciparum* MSP_1-19_. Expression of *Chlamydomonas* GBSS-*Plasmodium* fusion protein is driven by a strong chimeric Rubisco *RBCS2* and *HSP70A* promoter. (**b**) High-level of GBSS-*P. falciparum* MSP_1-19_ protein expression in one representative *Chlamydomonas* transformant (P5) was obtained by co-transformation of the BafR1 mutant strain lacking the *GBSS* gene. Samples representing 1 mg of purified starch after French press disruption and Percoll-gradient centrifugation were resuspended in SDS-βmercaptoethanol loading buffer, separated by SDS-PAGE and stained by Coomassie blue. Note that minor protein bands accidently trapped in the polysaccharide matrix are common to wild type WT, P5 and mutant BafR1 algae lacking the GBSS gene. The starch protein extracts from WT and P5 algae were also blotted to nitrocellulose and incubated with rabbit polyclonal antibodies specific to *C. reinhardtii* GBBS (**c**), or rabbit anti-*P. falciparum* MSP_1-19_ (**d**).The molecular weights of protein markers are given in kDa.

We constructed a *Chlamydomonas* expression vector containing the gene encoding a GBSS protein carrying a deletion that yielded a product truncated for 130 amino acids at the C-terminus (Figure 1a and Figure S1 online). We have previously shown that the absence of this C-terminal tail does not impair stability and targeting into the chloroplast [15]. A synthetic gene that encoded the 19 kDa C-terminal peptide of *P. falciparum* MSP1 (*Pf*MSP_1-19_) was designed taking into account the GC-rich codon bias of *Chlamydomonas* (Figure S2). This synthetic gene was fused to the truncated *GBSS* gene followed by the paromomycin resistance gene for selection of algal transformants (Figure 1a). The expression of GBSS-*Pf*MSP_1-19_ fusion protein is under the control of a strong chimeric rubisco *RBCS2* and *HSP70A* promoter (Figure 1a). The *Chlamydomonas* BafR1 mutant strain with a complete gene deletion at the *STA2* locus that encodes GBSS [18] was transformed with the GBSS-*Pf*MSP_1-19_ expression vector. The transformants developed normally during vegetative growth and were indistinguishable from wild type algae. Starch granules from *Chlamydomonas* transformants were purified using French press disruption followed by sedimentation and Percoll gradient centrifugation. The starch bound proteins were analyzed on SDS-PAGE and Coomassie blue staining. Out of twenty clones analyzed in a pilot experiment, three *Chlamydomonas* transformants strongly expressed the GBSS-*Pf*MSP_1-19_ fusion, as determined by their expected electrophoretic mobility. One positive *Chlamydomonas* transformant, designated P5, showed levels of starch-associated GBSS-*Pf*MSP_1-19_ protein that are slightly higher than those of the standard GBSS of wild type reference algae (Figure 1b **,** lanes P5 and WT). This increase of GBSS-*Pf*MSP_1-19_ in transgenic algae compared to the wild type GBSS is probably due to both the chromosomal insertion site insertion and the strength of the chimeric HSP70A/RbcS2 promoter used in this context. In addition, the promoter of the construct might not respond in an identical fashion to circadian clock control, which is known to operate on the wild-type GBSS gene [23]. Western blots using rabbit-specific polyclonal antibodies confirmed that the fusion protein of P5 transgenic algae contains the *Chlamydomonas* starch bound GBSS fused to the *P. falciparum* MSP_1-19_ antigen (Figure 1c and 1d).

### Demonstration that genetically engineered starch particles stored malaria antigens inside the chloroplast of *Chlamydomonas*

**Figure 2:**
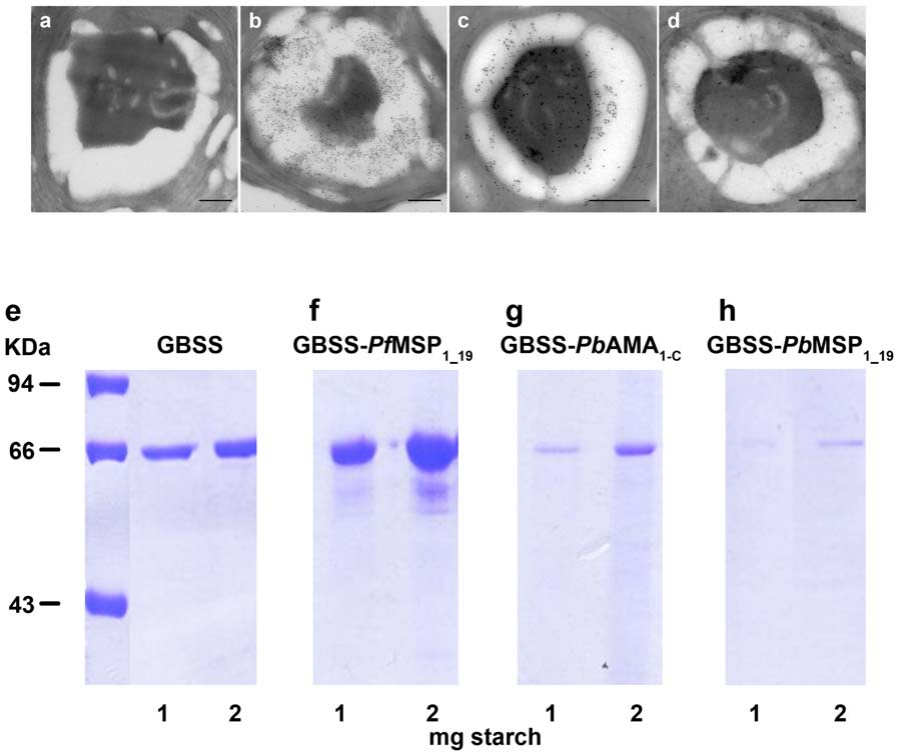
Malarial antigen accumulation in starch of transgenic *C. reinhardtii*. Pre-immune rabbit serum used as control shows no gold particles either on starch grains or over the pyrenoid matrix (**a**). Transverse sections of transgenic algae visualized by electron microscopy and immunogold labelling with rabbit polyclonal antibodies specific to *P. falciparum* MSP_1-19_ (**b**), *P. berghei* AMA_1-C_ (**c**) and *P. berghei* MSP_1-19_ (**d**). Note the presence of positive gold particles (black dots) on the starch grains (white) surrounding the pyrenoid matrix in the middle, which also contained a significant amount of GBSS-parasite fusion protein. The bar represents 500 nm. The total starch-bound protein content of purified granules containing wild type GBSS (**e**) was compared to those of GBSS-*Pf*MSP_1-19_ (**f**), GBSS-*Pb*AMA_1-C_ (**g**) and GBSS-*Pf* MSP_1-16_ (**h**). One and two milligrams of starch purified from transformants and wild type algae were analyzed by SDS-PAGE and stained by Coomassie blue. The molecular weights of three protein markers are given in kDa.

While we explored the expression of *P. falciparum* MSP_1-19_ in *Chlamydomonas*, we also extended our system to production of other malaria candidate vaccine antigens. AMA1 and MSP1 C-terminal domains derived from *P. berghei*, designated GBSS-*Pb*AMA_1-C_ and GBSS-*Pb*MSP_1-19_ (Figure S2), were investigated in detail, as the murine malaria parasite is frequently used for animal vaccine formulation and testing. *Chlamydomonas* transformed with these constructs were screened by visualising the starch bound proteins among a sample of 20 to 200 transformants. The presence of transgenic starch containing GBSS-*Pb*AMA_1-C_ and GBSS-*Pb*MSP_1-19_ was demonstrated by ultrastructural-immunogold labelling using two positive algal clones and specific polyclonal antibodies (Figure 2c and 2d). As expected, there was no gold-staining observed by electron microscopy on the starch of wild type algae (Figure 2a), while strongly positive starch granules containing *P. falciparum* GBSS-*Pf*MSP_1-19_ were detected in transgenic algae (Figure 2b). The data suggest a stronger accumulation of *P. falciparum* GBSS-*Pf*MSP_1-19_ protein in the transgenic algae (Figure 2b) compared to *P. berghei* GBSS-*Pb*AMA _1-C_ and GBSS-*Pb*MSP_1-19_ fusion proteins (Figure 2c and 2d). The differences in levels of *P. berghei* GBSS-*Pb*AMA _1-C_ and GBSS-*Pb*MSP_1-19_ fusion proteins relative to that of *P. falciparum* GBSS-*Pf*MSP_1-19_ are likely due mainly to the influence of random site integration events in the chromosomes of transgenic *Chlamydomonas.* Hence, all GBSS-*Chlamydomonas* transformants expressed the starch-associated GBSS-parasite antigens in transgenic algae chloroplasts, as illustrated by full-ultrastructural section (Figure S3). Generally, a significant accumulation of GBSS-*Pf*MSP _1-19,_ GBSS-*Pb*AMA_1-C_ and GBSS-*Pb*MSP_1-19_ was detected within the starch sheath around the single chloroplast pyrenoid (P) (Figure 2b-d and Figure S3), which defines a protein agglomerate of mostly Rubisco protein. However, gold particles were also observed over the pyrenoid structure itself. The immunogold signal over the pyrenoid was not detected in untransformed controls (Figure 2a), suggesting the presence of small fragments of transgenic starch granules surrounding the pyrenoid. The total recombinant GBSS-parasite fusion protein content was determined in all transgenic transformants after starch purification. The highest concentrations were confirmed in starch-associated GBSS-*Pf*MSP_1-19_ (Figure 2f), demonstrating the presence of higher levels of starch-bound protein in the strains expressing GBSS-*Pf*MSP_1-19_ in comparison to those expressing wild type GBSS alone (Figure 2e), GBSS-*Pb*AMA_1-C_ (Figure 2g), or GBSS-*Pb*MSP_1_19_ (Figure 2h). These results indicate that levels of starch associated GBSS-parasite fusion protein estimated at about 0.2 to 1.0 µg of protein per 1.0 mg of purified starch can be obtained, validating the usefulness of *C. reinhardtii* as an expression platform for complex recombinant parasite proteins within starch granules.

### Transgenic starch-bound antigens elicit immune responses and protection against *P. berghei* challenge

**Figure 3:**
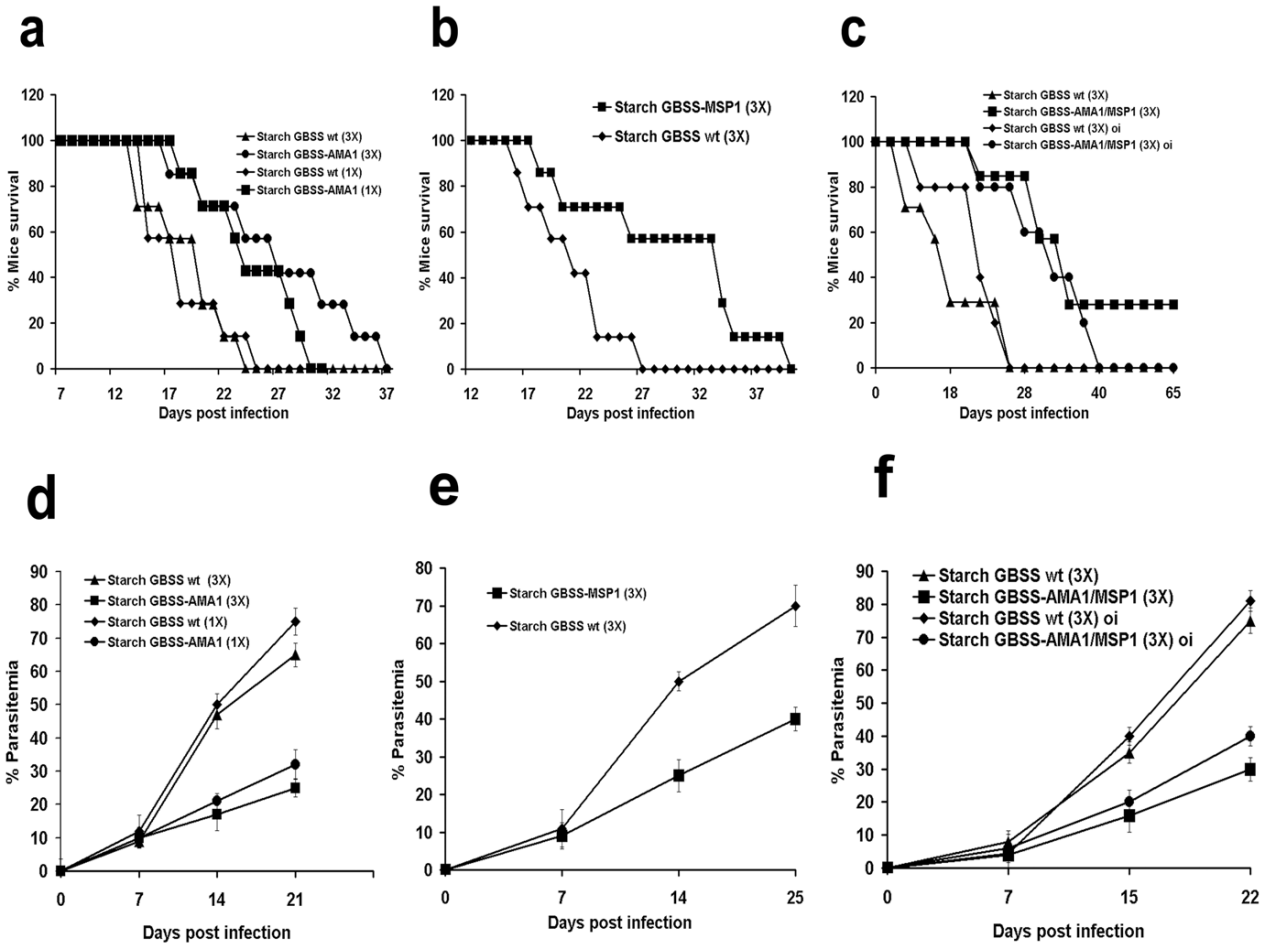
Starch-bound antigens elicit protection against *P. berghei* challenge. All mice survival experiments were performed using a group of 10 Balb/c mice. (**a**) Life expectancy of a group of mice vaccinated after a single, or three doses of starch containing GBSS-*Pb*AMA_1-C_. Each dose contains 10 mg of purified starch derived GBSS-*Pb*AMA_1-C_ and Freund's adjuvant. As a negative control, a group of mice was vaccinated with a single, or three doses of wild type (WT) starch containing GBSS alone. After immunization, mice were challenged with 10^4^ (lethal dose) of *P. berghei* ANKA strain. Four independent experiments have been performed (n = 4) and P<0.001. (**b**) Life expectancy of a group of mice immunized by three doses of starch containing GBSS-*Pb*MSP_1-19_. Each dose contains 10 mg of purified starch derived GBSS-*Pb*MSP_1-19_ and Freund's adjuvant. As a negative control, a group of mice was vaccinated with three doses of wild type (WT) starch containing GBSS alone. After immunization, the vaccinated mice were challenged with *P. berghei* and analyzed as above. Three independent experiments have been performed (n = 3) and P<0.05. (**c**) Life expectancy of a group of mice vaccinated by three doses of starch containing a mixture of 5 mg of GBSS-*Pb*AMA_1-C_ and 5 mg of GBSS-*Pb*MSP_1-19_ with Freund's adjuvant. As a negative control, a group of mice was also vaccinated with three doses of wild type (WT) starch containing GBSS alone. After vaccination, mice were challenged with *P. berghei* and analyzed as above. Three independent experiments have been performed (n = 3) and P<0.001. A group of mice were also fed three times with both starches GBSS-*Pb*AMA_1-C_ and GBSS-*Pb*MSP_1-19_ mixed with the B-subunit enterotoxin mucosal adjuvant. As a negative control, a group of mice was fed with wild type (WT) starch containing GBSS alone. After oral immunization, mice were challenged with 10^4^ (lethal dose) of *P. berghei* and analyzed as above. n = 3 and P<0.001. For all experiments, life expectancy and parasitemia were monitored daily. The panels **d**, **e** and **f** represent parasitemia profiles corresponding to mice vaccinated and challenged in experiments shown in panels **a**, **b** and **c**, respectively. Data represent mean values +/− s.d. and are derived from at least three or four independent experiments with similar results (P<0.001).

**Figure 4:**
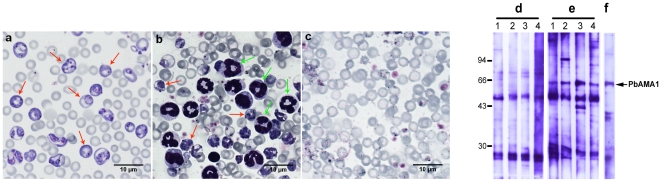
Starch-bound antigens elicit immune responses and reduce parasitemia after *P. berghei* challenge. The Giemsa stained thin smears of red blood cells isolated from mice vaccinated with starch containing wild type GBSS (negative controls), challenged with *P. berghei* ANKA and analyzed after 3 weeks post infection. The red arrows indicate that the mice were highly infected with *P. berghei* (**a**). The Giemsa stained thin smears of red blood cells isolated from mice vaccinated with starch containing both GBSS-*Pb*AMA_1-C_ and GBSS-*Pb*MSP_1-19_, challenged with *P. berghei* ANKA and analyzed after 3 weeks post infection (**b**). The Giemsa stained thin smear of red blood cells isolated from mice vaccinated with starch containing both GBSS-*Pb*AMA_1-C_ and GBSS-*Pb*MSP_1-19_, challenged with *P. berghei* ANKA and analyzed after 6 weeks post-infection (**c**). Note the presence of numerous leukocytes (probably neutrophils, green arrows) and fewer infected red blood cells (red arrows) in the vaccinated mice after 3 weeks post-infection (**b**). Both leukocytes and *P. berghei* infected red blood cells were not detected in mice, which survived after 6 weeks post-infection (**c**). Western blots of total extracts of antigens prepared from red blood cells infected by *P. berghei*. The immunoblots were incubated with the immune sera isolated from 4 mice immunized with starch containing wild type GBSS (panel **d**, lanes 1-4). Blots probed with immune sera of 4 mice immunized with starch containing GBSS-*Pb*AMA_1-C_ (panel **e**, lanes 1–4). Lane **f** corresponds to the blots incubated with the positive rabbit polyclonal antibodies specific to *P. berghei* AMA1 produced in *E. coli*.

To investigate whether the levels of transgenic starch-parasite antigens achieved were sufficient to elicit protective immune responses, we tested mice immunized with purified starch containing GBSS-*Pb*AMA_1-C_, or GBSS-*Pb*MSP_1-19_. After immunization, mice were challenged with lethal doses (10^4^ infected red blood cells) of the highly virulent *P. berghei* ANKA strain. A single dose of intraperitoneal immunization with starch containing GBSS-*Pf*AMA_1-C_ showed a significant and reproducible delay of mortality in *P. berghei*-challenged mice, compared to mice immunized with wild type starch GBSS alone (Figure 3a). When the animals were immunized with three doses of starch containing GBSS-*Pb*AMA_1-C_ prior to *P. berghei* challenge, a longer delay in mortality was observed (Figure 3a). Similarly, mice immunized with three doses of starch containing GBSS-*Pb*MSP_1-19_ displayed significant protection and delay in mortality after parasite challenge (Figure 3b). Consistent with the delay in mortality, progression in the proportion of red blood cells infected (% parasitemia) in immunized and challenged mice was slower in comparison to control animals (Figure 3d and 3e). Moreover, we showed that the sera from immunized mice contain specific immunoglobulins (IgG) generated against the native *P. berghei* AMA1 (*Pb*AMA1) using Western blot analyses (Figure 4, panel e). When mice were simultaneously immunized with both starch bound GBSS-*Pb*AMA_1-C_ and GBSS-*Pb*MSP_1-19_, a delay in mice mortality persisted for a longer period of time (Figure 3c) and reduction in parasitemia (Figure 3f) was also observed. Interestingly, 25–30% of mice challenged with *P. berghei* survived (Figure 3c) after two months post-challenge even though few infected red blood cells can be visualized (Figure 4, panel c compare to panels a and b). We confirmed that the remaining infected red blood cells were not viable parasites because they were not able to develop any disease symptoms and intra-erythrocytic development in naive mice. At this time-point, the duration of the induced protection was also investigated by re-challenging vaccinated mice with lethal doses of *P. berghei.* Vaccinated mice displayed sterile protection, since no infected red blood cells or any mortality was observed. There was also a significant delay in mortality when mice were fed purified starch containing both GBSS-*Pb*AMA_1-C_ and GBSS-*Pb*MSP_1-19_ (Figure 3c).

### Immune sera and purified IgG specific to starch bound antigen block red blood cell entry by *P. falciparum*

**Figure 5:**
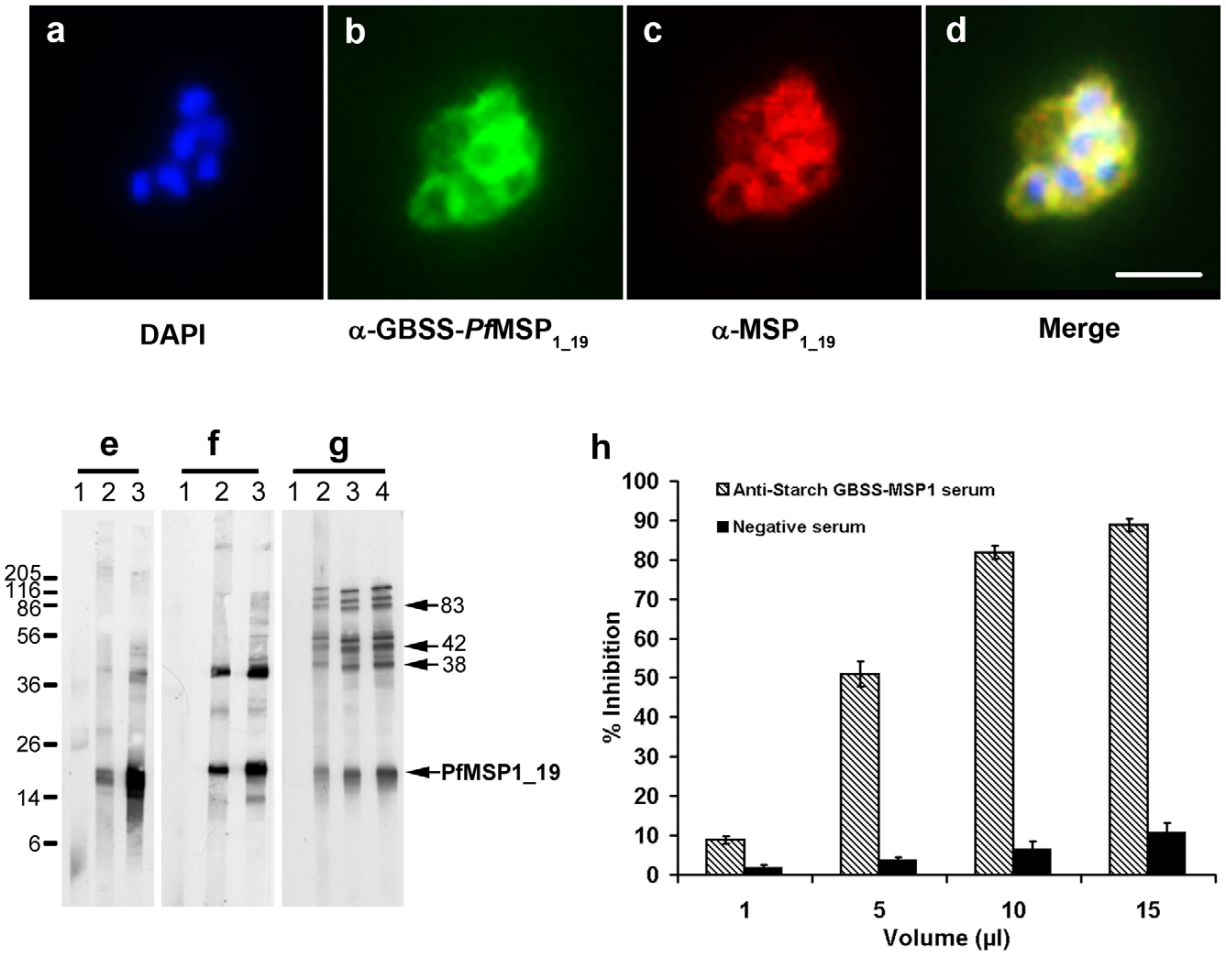
Inhibition of red blood cell entry by *P. falciparum*. Immunofluorescence assay (IFA) of erythrocyte-containing *P. falciparum* schizont with merozoites incubated with a pool of immune sera generated against purified starch GBSS-*Pf*MSP_1-19_ (**b**). The nuclei of merozoites were stained by DAPI (**a**). The same schizont was also stained with rabbit polyclonal antibodies specific to native MSP_1-19_ (**c**). Note the perfect overlap of fluorescence signals (**d,** yellow signal), which corresponds to merged pictures in panels **a**, **b** and **c**) of anti-starch GBSS-*Pf*MSP_1-19_ (**b,** green signal) and that of the positive control sera (red, **c**). The bar represents 5 µm. Western blots of total extract antigens from ring (**e**), trophozoite (**f**) and schizont (**g**) obtained after synchronization of *P. falciparum* culture with sorbitol and separated by polyacrylamide gel, transferred to nitrocellulose and probed with antibodies. Lane 1, a pool of non immune serum used as negative control. Lanes 2 and 3, a pool of immune sera from two independent experiments in which a group of mice were immunized with starch GBSS-*Pf*MSP_1-19_. Lane 4 in panel **g** is from a third independent experiment. The proteolytically-processed fragments of 83, 42, 38 and 19 kDa derived from the 185–205 kDa *Pf*MSP1 were shown by arrows. (**h**) Comparison of *in vitro* inhibitory effect of immune sera obtained after immunization with starch containing GBSS-*Pf*MSP_1-19_ with that of immune sera generated against starch containing wild type GBSS only using the deadly human *P. falciparum* HB3 strain. Histograms represent mean values +/− s.d. of separate experiments (n = 3). Results were confirmed in two additional independent experiments (P<0.001).

Humans are the natural host for *P. falciparum* and the paucity of animal models for infections with this *Plasmodium* species makes the discovery of vaccine candidates challenging. *Pf*MSP1 is a leading anti-blood stage malaria vaccine candidate [24], [25] that undergoes proteolytic processing during merozoite maturation resulting in four major fragments of 83, 30, 38 and 42 kDa [26]. Before erythrocyte entry, the 42-kDa fragment undergoes a secondary proteolytic cleavage, leaving the C-terminal 19-kDa fragment (MSP_1-19_) associated with the merozoite [24]–[26]. Antibodies that are specific for MSP_1-19_ in naturally exposed individuals have potent inhibitory activities against *P. falciparum* growth *in vitro* [24]. To explore whether antibodies elicited by starch-associated antigens could inhibit *in vitro* growth of *P. falciparum,* GBSS-*Pb*MSP_1-19_ starch was used to immunize a group of ten mice. Indirect immunofluorescence assays were used to demonstrate that antibodies, which were raised against purified starch containing GBSS-*Pf*MSP_1-19_ recognized specifically *P. falciparum* merozoites within mature schizonts, as shown by co-localization with the signal of native MSP1 detected with rabbit specific antibodies (Figure 5a-d). In addition, the anti-GBSS-*Pf*MSP_1-19_ antibodies recognized all *Pf*MSP1 processed products including the major 19-kDa merozoite surface antigen (Figure 5e-5g). Importantly, anti-GBSS-*Pf*MSP_1-19_ specific sera displayed very strong inhibition (circa 95%) of red blood cell invasion by *P. falciparum* (Figure 5h)**.** A dose-dependent inhibition of red blood cell invasion by *P. falciparum* was also seen with IgG purified from sera raised against starch-bound GBSS-*Pf*MSP_1-19_ (Figure S4). Our data indicate that 50% of growth inhibition of *P. falciparum* HB3 strain can be achieved with approximately 7+/−0.5 µg/ml (P<0.005) of purified IgG while a weaker inhibition was observed with the 3D7 strain. These results demonstrate that the biological activities of the antibodies produced against starch associated GBSS-*Pf*MSP_1-19_ can be obtained in a parasite strain-specific manner [27].

### Conclusion

We have engineered high levels of starch-bound GBSS fused to three distinct malaria vaccine candidates using the unicellular green algae *C. reinhardtii.* Our animal vaccine studies suggest that the levels of transgenic starch antigens that accumulate in the chloroplast are sufficient to confer substantial protection against lethal *P. berghei* in mice. Additionally, the functional activity of antibodies elicited to *P. falciparum* was demonstrated by inhibition of parasite growth *in vitro* and we ascribed this to blockade of erythrocyte invasion. *C. reinhardtii* thus provides an ideal expression system for the production of recombinant antigens associated with the starch polysaccharide matrix. In addition, algae can be grown on scales ranging from a few millilitres to 500,000 liters. Starch metabolism has been demonstrated to be identical in *C. reinhardtii* to that found in vascular plants so that results obtained are likely to be immediately transposable to crop plants including cereals and potatoes [28], [29]. Aside from the tremendous advantage of producing clinically relevant antigens in starch, transgenic algae can also be generated quickly, requiring only a few weeks from the generation of initial transformants to large scale vaccine production. These attributes, and the fact that unicellular green algae fall into the GRAS (Generally Regarded As Safe) category, make *C. reinhardtii* a particularly attractive alternative for the expression of recombinant antigens. Moreover, starch is easy to purify and represents a protective environment for bound proteins because GBSS is known to be exceptionally stable with no detectable loss of activity even after years of storage. In a first approach to genetically engineering starch particles designated amylosomes, we have produced recombinant anti-malaria vaccines in starch purified from *C. reinhardtii*. This system should also be amenable to the production of virtually any recombinant antigens including vaccine candidates of viruses, bacteria and other protozoan parasites.

## Materials and Methods

### Plasmid construction and *Chlamydomonas* transformation

The pKB101 vector used to transform *Chlamydomonas* for expression of *Plasmodium* antigens in the chloroplast-starch compartment is derived from the previously described pMS188 plasmid [30]. The bleomycin resistance gene flanked by *MscI* and *BamHI* restriction sites, which is under the control of chimeric *RBCS2* and *HSP70A* promoters was replaced by a PCR product corresponding to a truncated version of the genomic sequence (*STA2*) [15], encoding the GBSS protein lacking the 130 C-terminal amino-acids. The 5′ end of this PCR fragment (3 kb) was cloned blunted while the 3′ end was modified by inserting into the primer both *XhoI* and *BamHI* restriction sites. These sites were used to clone in-frame all *Plasmodium* synthetic cDNAs designed based on the codon bias of *C. reinhardtii*. The *AphVIII* gene conferring paromomycin resistance was introduced for efficient selection of nuclear *Chlamydomonas* transformants. The *AphVIII* gene was obtained by PCR amplification from the pSL18 plasmid [31]. The different expression vectors used in this study were introduced by nuclear transformation using the glass beads method [32] into the cell wall-less GBSS mutant strain of *Chlamydomonas* BafR1 [18]. The transformants were selected on TAP medium plates [33] supplemented with paromomycin at 10 µg.mL^−1^. The strains expressing the chimeric proteins were identified by analyzing the pattern of proteins bound to the starch granule by SDS-PAGE and Coomassie blue staining.

### Electron microscopy, immunofluorescence assays and immunoblot analyses

For immuno-electron microscopy, the pellets of transformed and wild type *Chlamydomonas* were fixed overnight at 4°C in 8% paraformaldehyde in PBS buffer, thoroughly washed in the same buffer and infused in 2.3 M sucrose containing 20% polyvinyl pyrrolidone 10000 in 0.1 M phosphate buffer. The pellets were mounted on ultracryotome supports and rapidly frozen in liquid nitrogen. Ultrathin sections of about 90 nm were obtained and the grids were incubated with rabbit polyclonal antibodies specific to *P. falciparum* MSP1, *P. berghei* MSP1 (generously supplied by Dr Tony Holder, Mill Hill, UK) or *P. berghei* AMA1 (provided by Dr. Christopher Adda, La Trobe University, Australia). After washing, the sections were incubated at room temperature for 30 min in the corresponding secondary gold conjugates (Jackson ImmunoResearch Laboratories Inc.). After staining with 0.5% uranyl acetate in 1.5% methyl cellulose, the sections were observed on a Hitachi H600 transmission electron microscope.

For immuno-fluorescence assays (IFA), intracellular parasites were permeabilized with 0.1% Triton X-100 in PBS containing 0.1% glycine (PBS-T) for 10 minutes at room temperature. Samples were blocked with 3% BSA in PBS-T buffer and mice immune sera diluted at 1∶1000 were added on parasites in the same buffer for one hour at 37°C. Rabbit secondary antibody coupled to Alexa-488 nm (Molecular Probes) diluted at 1∶1000 was added in addition to DAPI for nucleus staining. For co-localization assay, the rabbit anti-MPS1 serum raised against bacterial expressed recombinant *Pf*MSP1 protein and the goat secondary antibody coupled to Alexa 500 nm were used at the same dilution. Fluorescence was visualized with a ZEISS Axiophot microscope.

For immunoblot analyses, total protein extracts from the synchronized *P. falciparum* HB3 strain corresponding to ring, trophozoite or schizont stage, respectively were used. The asynchronous *P. berghei* parasitized red blood cells isolated from mice or purified starch containing GBSS-*P. berghei* antigens were also analyzed. The samples were boiled in Laemmli's buffer, separated by SDS-PAGE and transferred to Hybond ECL nitrocellulose (Amersham).

### *In vitro* inhibition of red blood cell entry by *P. falciparum* in the presence of immune sera and purified IgG

The *P. falciparum* HB3 or 3D7 strain was grown in O^+^ red blood cells at 6% hematocrit in RPMI 1640 medium supplemented with 10% of AB^+^ human serum. Cultures were maintained at 37°C and in 90% N_2_, 5% CO_2_, 5% O_2_ atmosphere. Synchronized parasites were obtained by two sorbitol treatments at a 32-hour interval. Synchronized infected red bloods containing schizonts with 1% parasitemia at 2% hematocrit using fresh red blood cells were cultured in complete RPMI medium and invasion assays were performed as previously described [34]. For standardized growth inhibition assays (GIA) validated in the Laboratory of Malaria and Vector Research (NIAID/NIH, USA), IgGs from mice immunized with starch containing GBSS-*Pf*MSP_1-19_ were purified and tested as previously described [22].

### Mouse immunization with transgenic starch and *P. berghei* challenge

Female BALB/c of 6 weeks of age were purchased and kept in the Pasteur Institute facility. All animals were fed with regular diet and all procedures were in accordance with national regulations on animal experimentation and welfare authorized by the French Ministry of Agriculture and Vetenary committee (N° 59-009145). The Pasteur Institute of Lille and the CNRS review board or ethics committee specifically approved this study. A group of 10 mice were intraperitoneally immunized with complete Freund's adjuvant and three weeks later were challenged twice with incomplete adjuvant in two-week intervals using 10 mg (per mouse) of starch containing GBSS-*Pb*AMA_1-C_ and/or GBSS-*Pb*MSP_1-19_ or fed three times with the same material in the presence of B-subunit of heat-labile enterotoxin (LTB) from *E. coli* (Sigma). In some experiments, a single dose of vaccination was performed. Starch containing wild type GBSS alone (10 mg per mouse) was used to immunize intraperitoneally or orally 10 mice for negative vaccination controls. The vaccinated mice were challenged with 10,000 red blood cells parasitized by *P. berghei*. Parasite growth in vaccinated mice was monitored by Giemsa-stained thin blood smears, and survival of mice was assessed daily.

### Statistical analysis

Statistical differences between groups of mice used in this study were evaluated by the Student's t-test. The Mann-Whitney test was used for morphometric Giemsa-staining data and the Log Rank test for survival curves.

## Supporting Information

Figure S1 **Nucleotide sequences of** ***C. reinhardtii*** **plasmid.** The plasmid pKB101 was used to clone *P. falciparum* and *P. berghei* genes. This *C. reinhardtii* expression vector contains the chimeric *RBSC2*-*HSP70A* promoter (nucleotide 796 to 1040), GBSS genomic DNA sequence deleted at the 3' end for the 390 last nucleotides (nt 1048 to 4104), blaM Beta-lactamase also named ampicillin resistance cassette (nt 5137 to 5995), ColE1 ori (nt 6155 to 6748), AphVIII or paromomycin resistance cassette (7234-9259).
(TIF)

Figure S2 **Parasite nucleotide sequences used for cloning and expression of** ***P. falciparum*** **MSP_1-19_,** ***P. berghei*** **MSP_1-19_ and** ***P. berghei*** **AMA_1-C._** The nucleotide sequences of *Plasmodium* genes were designed according to the codon bias of *C. reinhardtii*.
(TIF)

Figure S3 **Longitudinal section of** ***C. reinhardtii*** **cell was visualized by electron microscopy.** The section of *C. reinhardtii* cell was probed with polyclonal antibodies specific to *P. berghei* AMA1. The algae cell has been transformed by a construct expressing *P. berghei* AMA_1-C_ antigen in the starch localized in the chloroplast. S represents starch grains surrounding the pyrenoid (P) matrix. The gold particles bind to specific anti-AMA1 antibodies present on both starch and the pyrenoid matrix. N, nucleus; C, chloroplast; M, mitochondrion. The bar  = 500 nm.
(TIF)

Figure S4 ***P. falciprum*** **strain-specific growth inhibitory effects in the presence of purified IgGs.** A pool of immune sera of mice vaccinated with starch containing GBSS-*Pf*MSP_1-19_ was used to purify IgGs. The inhibition of red blood cell invasion by either *P. falciparum* HB3 or 3D7 strain was tested in the presence of purified immune IgGs. Data represent mean values +/- s.d. and are from at least three independent experiments with two different pools of immune sera (P<0.005).
(TIF)
