# Supplementary figures and images for: Engineering the Chloroplast Targeted Malarial Vaccine Antigens in Chlamydomonas Starch Granules
Source: PLoS One. 2010 Dec 15;5(12):e15424. doi: 10.1371/journal.pone.0015424 (PMC3002285; doi:10.1371/journal.pone.0015424)

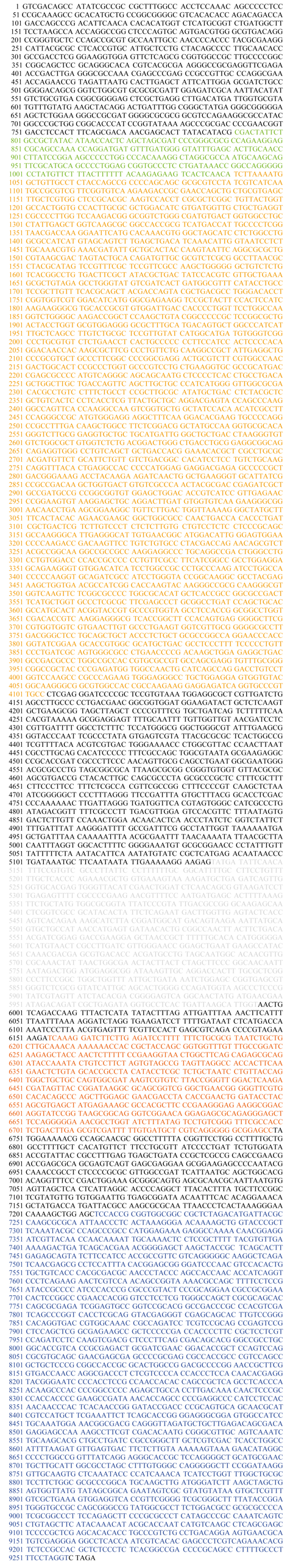

Supplement: Figure S1 — Nucleotide sequences of C. reinhardtii plasmid. The plasmid pKB101 was used to clone P. falciparum and P. berghei genes. This C. reinhardtii expression vector contains the chimeric RBSC2-HSP70A promoter (nucleotide 796 to 1040), GBSS genomic DNA sequence deleted at the 3' end for the 390 last nucleotides (nt 1048 to 4104), blaM Beta-lactamase also named ampicillin resistance cassette (nt 5137 to 5995), ColE1 ori (nt 6155 to 6748), AphVIII or paromomycin resistance cassette (7234-9259). (TIF) [file pone.0015424.s001.tif]

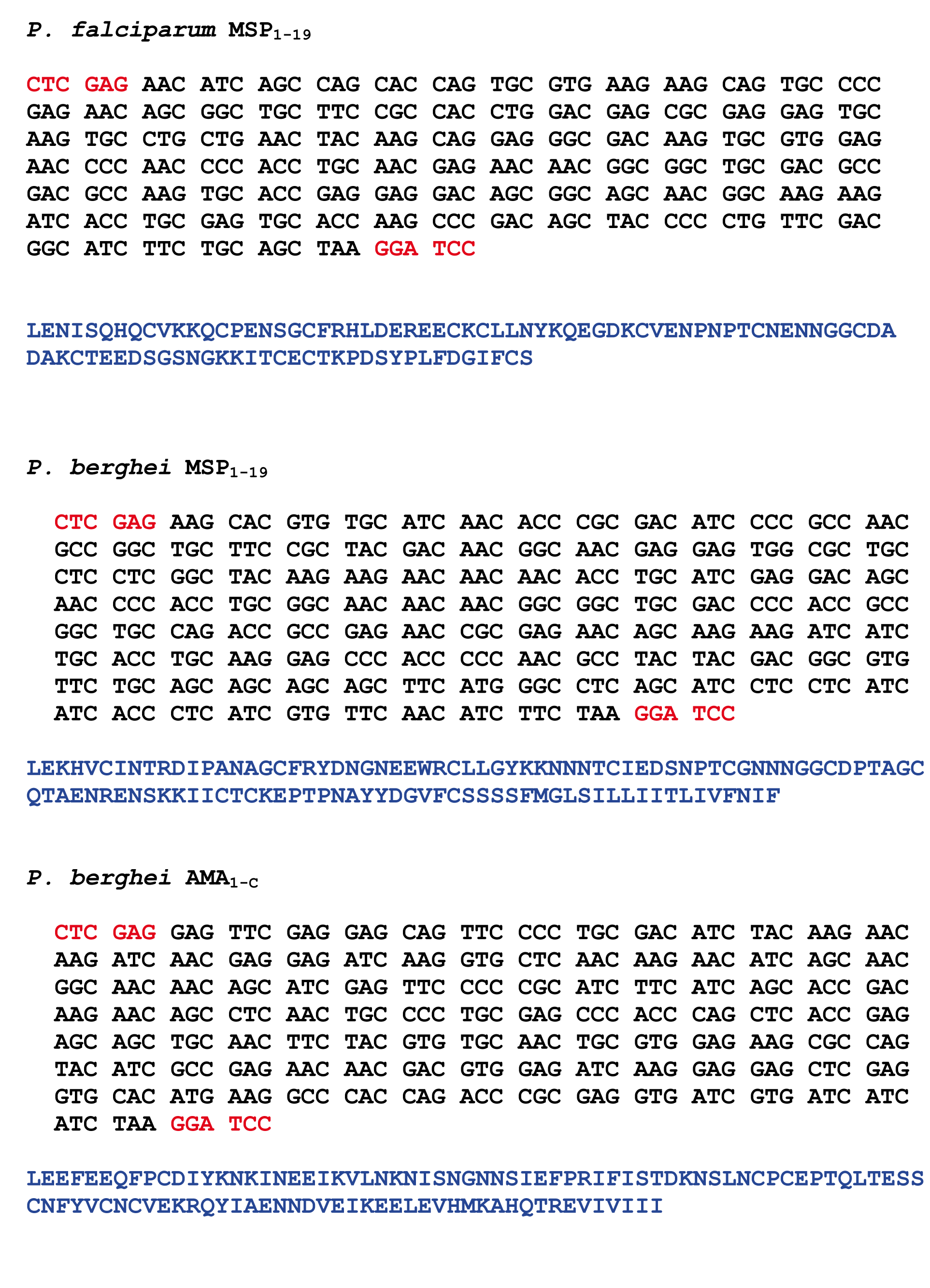

Supplement: Figure S2 — Parasite nucleotide sequences used for cloning and expression of P. falciparum MSP1-19, P. berghei MSP1-19 and P. berghei AMA1-C. The nucleotide sequences of Plasmodium genes were designed according to the codon bias of C. reinhardtii. (TIF) [file pone.0015424.s002.tif]

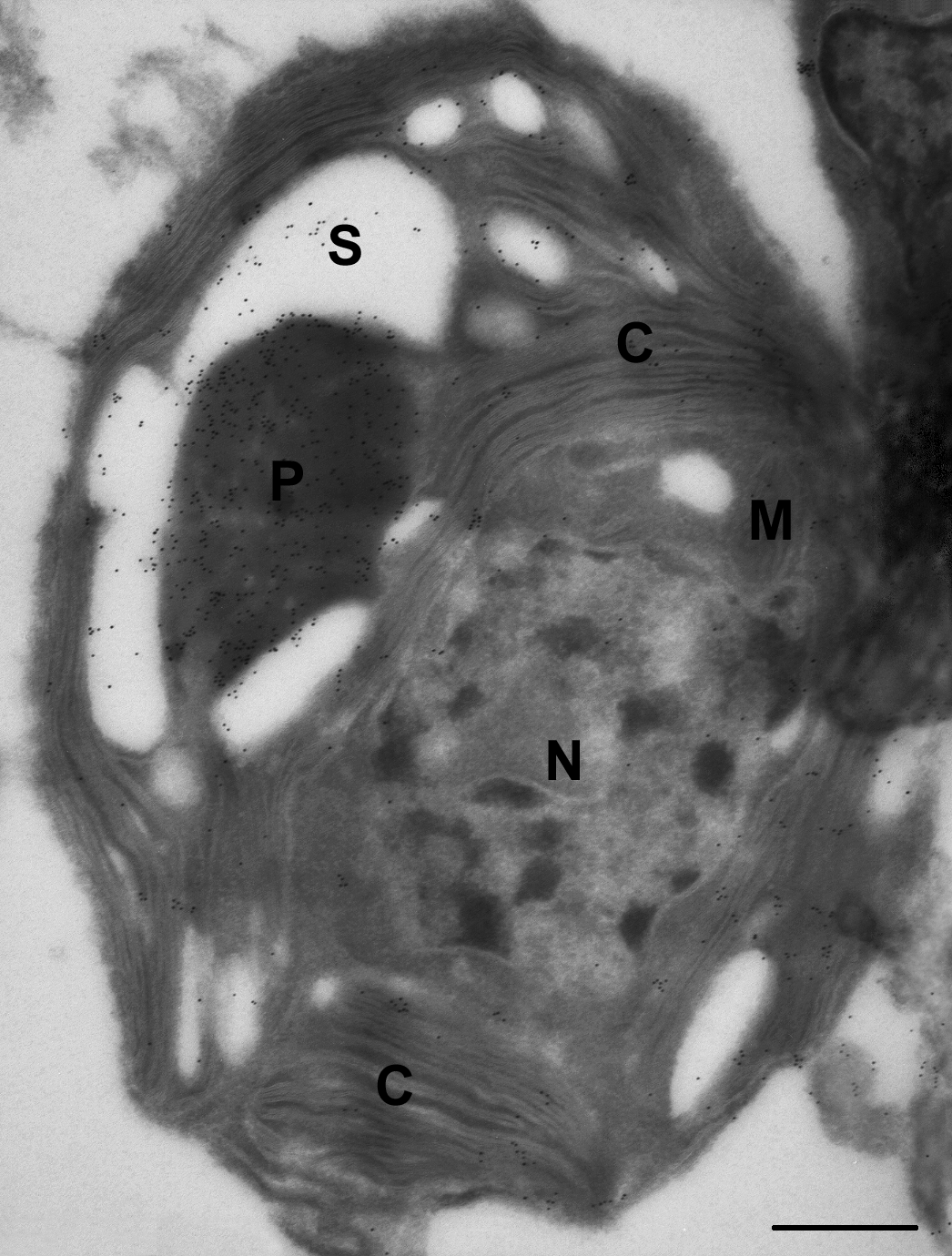

Supplement: Figure S3 — Longitudinal section of C. reinhardtii cell was visualized by electron microscopy. The section of C. reinhardtii cell was probed with polyclonal antibodies specific to P. berghei AMA1. The algae cell has been transformed by a construct expressing P. berghei AMA1-C antigen in the starch localized in the chloroplast. S represents starch grains surrounding the pyrenoid (P) matrix. The gold particles bind to specific anti-AMA1 antibodies present on both starch and the pyrenoid matrix. N, nucleus; C, chloroplast; M, mitochondrion. The bar = 500 nm. (TIF) [file pone.0015424.s003.tif]

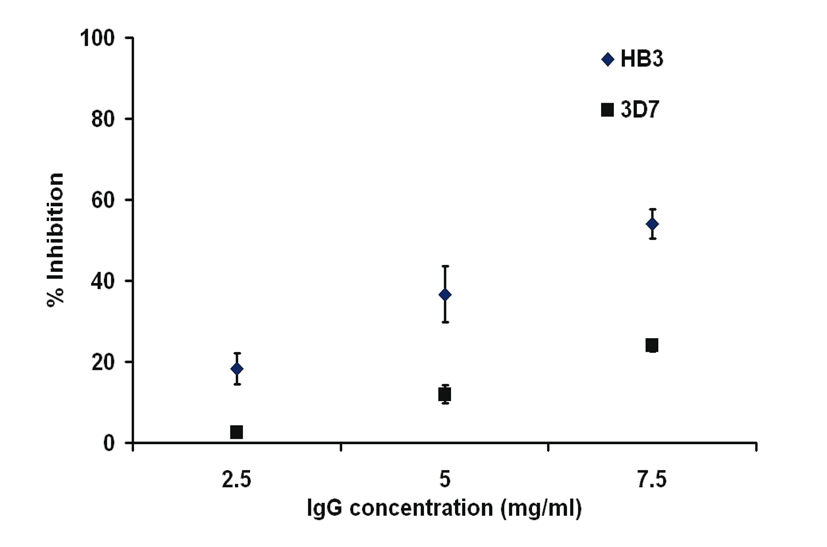

Supplement: Figure S4 — P. falciprum strain-specific growth inhibitory effects in the presence of purified IgGs. A pool of immune sera of mice vaccinated with starch containing GBSS-PfMSP1-19 was used to purify IgGs. The inhibition of red blood cell invasion by either P. falciparum HB3 or 3D7 strain was tested in the presence of purified immune IgGs. Data represent mean values +/- s.d. and are from at least three independent experiments with two different pools of immune sera (P<0.005). (TIF) [file pone.0015424.s004.tif]
